# Non-Steroidal Anti-Inflammatory Drug Use and Genomic DNA Methylation in Blood

**DOI:** 10.1371/journal.pone.0138920

**Published:** 2015-09-22

**Authors:** Lauren E. Wilson, Sangmi Kim, Zongli Xu, Sophia Harlid, Dale P. Sandler, Jack A. Taylor

**Affiliations:** 1 Epidemiology Branch, National Institute of Environmental Health Sciences, National Institutes of Health, Research Triangle Park, North Carolina, United States of America; 2 Section of Hematology/Oncology, Department of Medicine, Georgia Regents University Cancer Center; Medical College of Georgia, Augusta, Georgia, United States of America; Emory University, UNITED STATES

## Abstract

**Background:**

Non-steroidal anti-inflammatory drug (NSAID) use is associated with decreased risk of some cancers. NSAID use modulates the epigenetic profile of normal colonic epithelium and may reduce risk of colon cancer through this pathway; however, the effect of NSAID use on the DNA methylation profile of other tissues including whole blood has not yet been examined.

**Findings:**

Using the Sister Study cohort, we examined the association between NSAID usage and whole genome methylation patterns in blood DNA. Blood DNA methylation status across 27,589 CpG sites was evaluated for 871 women using the Illumina Infinium HumanMethylation27 Beadchip, and in a non-overlapping replication sample of 187 women at 485,512 CpG sites using the Infinium HumanMethylation450 Beadchip. We identified a number of CpG sites that were differentially methylated in regular, long-term users of NSAIDs in the discovery group, but none of these sites were statistically significant in our replication group.

**Conclusions:**

We found no replicable methylation differences in blood related to NSAID usage. If NSAID use does effect blood DNA methylation patterns, differences are likely small.

## Background

Non-steroidal anti-inflammatory drugs (NSAIDs) are commonly used agents which reduce inflammation by inhibiting the cyclooxygenase pathway’s production of various prostaglandins from arachadonic acid. This pathway is a key part of the inflammatory response. Epidemiologic studies indicate that long-term NSAID usage is associated with decreased risk of certain cancers including including colorectal cancer, gastric cancer, and potentially breast cancer [1–4]. The biological mechanisms of chemoprevention related to this drug class remain unclear.

In animal models of colorectal cancer development, chronic inflammation is linked to accumulation of DNA methylation changes over time; this may affect epigenetic control of gene expression [5, 6]. These observed methylation changes may occur in the process of repairing damage caused by inflammation; *in vitro* studies have indicated that inducing oxidative stress and DNA damage in human cancer cell lines results in up-regulation and recruitment of DNA methyltransferases and other gene-silencing proteins to CpG islands [7, 8]. In some human cell lines, endogenously produced prostaglandins from the COX-2 pathway directly increased expression of DNA methyltransferases and influenced methylation of promoter regions of genes involved in epigenetic regulation [9, 10].

Long-term NSAID usage, which reduces systemic inflammation, is associated with differential methylation of certain genes in gastric and colon mucosa [11, 12]. These NSAID-linked methylation differences have only been investigated in a limited number of tissues; differences associated with long-term NSAID use have not been examined on the genomic level in blood. The inflammatory pathway targeted by NSAIDs downregulates the expression of enzymes responsible for maintence of DNA methylation [13], giving rise to the hypothesis that long term NSAID use may protect against aberrant genomic methylation changes associated with cancer.

## Methods

### Study population and data sources

We utilized data from the Sister Study, a prospective cohort of 50,884 women who had a sister with breast cancer but who did not have breast cancer themselves at enrollment [14]. All women provided detailed medication history at enrollment via a computer-assisted telephone interview. As NSAIDs are often used sporadically, we focused on women who used NSAIDs regularly. Women were defined as regular users if they reported taking an NSAID drug 3 or more times a week for at least 3 months. Regular NSAID users provided information on the types of NSAIDs taken and the frequency and duration of their use.

We examined the relationship between methylation and NSAID exposure using two methylation datasets within the Sister Study. The “discovery set” was comprised of 871 women with methylation data available on 27,589 CpG (cytosine-phosphate-guanine) sites from a nested case-cohort study that was designed to identify blood-based DNA methylation markers associated with breast cancer. A second, smaller”replication set” was comprised of 187 women with methylation array data on 485,512 CpG sites from a nested case-control study of diethylstilbestrol (DES) exposure. Study populations and details of methods for methylation data have been previously described [14, 15].

### Statistical Analysis

To examine the association between NSAID usage and DNA methylation, we used robust linear regression modeling to identify differential methylation of CpG sites for defined categories of NSAID usage compared to women who reported no regular lifetime use. The false discovery rate (FDR) was set at q<0.05 to correct for multiple testing; the correction method has been previously described [14, 16]. CpG sites passing the false discovery rate in the discovery set were then tested in the replication set for association between NSAID use and methylation. An association was considered replicated if the association p-value fell below a Bonferroni-corrected p-value (p ≤ 0.05 divided by the number of CpGs tested for replication). Data pre-processing, normalization methods and quality control measures are detailed in the supplementary material of our previously published papers [14, 16]. To summarize briefly, methylation intensity values were background-corrected using the Robust Multichip Average (RMA) method [17]and quantile-normalized across arrays. Methylation array plates included controls with known methylation levels to assess precision of measurement and duplicate samples to assess reproducibility of results within the assay. Each methylation array included probes to assess bisulfite conversion efficiency and negative control probes to measure background fluorescent intensity. Samples with poor bisulfite conversion efficiency (< 3,800) or having >5% of probes with unreliable measures (detection P > 0.05) were excluded. The methylation outcome was calculated using fluorescence intensities for unmethylated (U) and methylated (M) alleles as M/(M+U+100), which is a continuous variable ranging from 0 (completely unmethylated) to 1 (100% methylated) based on the ratio. In both data sets a nonspecific filtering step was applied to filter out the 20% CpGs with the smallest interquartile range (IQR) of methylation values before association analysis. CpG probes with single nucleotide polymorphisms (SNPs) present at target sites (428 CpGs from the HumanMethylation27 BeadChip and 20,869 CpGs from the HumanMethylation450 BeadChip) were excluded from the analysis. In the 27K data set we tested 21,659 probes and in the 450K data set we tested 369,120 probes.

To examine the association between long-term NSAID usage and DNA methylation, we used robust linear regression modeling to identify differential methylation of CpG sites for each defined strata of NSAID usage compared to a participant group with the lowest reported lifetime NSAID usage. Singular value decomposition (SVD) analysis of the raw dataset revealed that the top principal components derived from the methylation beta value matrix were highly correlated with plate, bisulfite conversion intensities and age. We adjusted for these factors together with breast cancer status in all association analyses. All association tests were also adjusted for the proportions of different types of white blood cells estimated using a method described by Houseman et al.[18, 19]

We conducted the robust linear regression modeling analyses separately in the breast cancer-free subcohort as well as in the entire study group with adjustment for breast cancer case status. We examined the effect of different durations of NSAID usage on methylation, as well as NSAID class, dosage and frequency of use. NSAID use and methylation analyses were conducted using SAS 9.3 (SAS Institute, Cary, NC, USA) and R (http://www.r-project.org.). Power calculations were conducted with Stata 13.1 (Statacorp, College Station, TX, USA).

### Ethics Statement

Informed written consent was obtained from all participants prior to participation. The study was approved by the Institutional Review Boards of the National Institute of Environmental Health Sciences (NIEHS), National Institutes of Health, and the Copernicus Group (http://www.cgirb.com/irb-services/).

## Results

All included participants were white, non-hispanic, and either had developed invasive breast cancer or were cancer free at the time of methylation analysis. 490 women reported ever regularly using NSAIDs in the discovery set of 871 participants (Table 1). Usage rates did not differ between cases and non-cases (56.4% and 56.2%). In our replication set, 54% reported regular NSAID use. Across cases and controls, users reported a mean regular lifetime NSAID use of approximately 7.7 years. Regular users reported a mean of 27.9 “pill-years” of use, which was calculated as the number of NSAID pills taken per week multiplied by the number of years that dose was taken. 329 women (37.8%) reported that they had taken an NSAID drug daily for at least 3 months within the past 12 months.

**Table 1:** Demographic characteristics of women in the discovery set (N = 871).

	All women(N = 871)		Breast cancer-free women (N = 573)		Breast cancer cases(N = 298)	
	N	(%)	N	(%)	N	(%)
**BMI**						
<25	371	(42.6)	245	(42.8)	126	(42.3)
25–29	226	(25.9)	153	(26.7)	73	(24.5)
29–35	159	(18.3)	98	(17.1)	61	(20.5)
35+	115	(13.2)	77	(13.4)	38	(12.7)
**Menopausal Status**						
Pre-menopausal	328	(37.7)	219	(38.2)	109	(36.6)
Post-menopausal	543	(62.3)	354	(61.8)	189	(63.4)
**Smoking Status**						
Never	458	(52.6)	294	(51.3)	164	(55.0)
Social	20	(2.3)	15	(2.6)	5	(1.7)
Past	331	(38.0)	222	(38.7)	109	(36.6)
Current	62	(7.1)	42	(7.3)	20	(6.7)
**Ever used an NSAID regularly** ^a^						
No	381	(43.7)	251	(43.8)	130	(43.6)
Yes	490	(56.3)	322	(56.2)	168	(56.4)
**Mean age at baseline**						
Mean (SD)	55.1	(9.1)	54.6	(8.9)	56.1	(9.2)
**Total years of use reported among users**						
Mean (SD)	7.7	(9.3)	7.8	(9.6)	7.6	(8.7)
**Total pill-years of use reported among all users**						
Mean (SD)	27.9	(68.7)	28.2	(67.7)	27.2	(70.6)
**Used an NSAID daily in the past 12 months**						
No	542	(62.2)	363	(63.4)	179	(60.1)
Yes	329	(37.8)	210	(36.6)	119	(39.9)

^a^used an NSAID > = 3 times a week for 3 or more months

In an analysis limited to women who did not develop breast cancer, no CpGs were differentially methylated between regular NSAID users and non-users, or when limited to NSAID users with recent daily use of the drugs. When examining years of NSAID use and pill-years of NSAID use as continuous exposures, we found 8 CpGs associated with pill-years of use that passed the FDR in the discovery set. However, only one of these CpGs reached p<0.05 in the replication set and failed to pass the Bonferroni correction.

When women who developed breast cancer during follow-up were included in the discovery set with adjustment for case status, we identified 122 CpG sites that were differentially methylated at FDR q<0.05 for ever-users of NSAIDs compared to never-users (Table 2). When we examined these sites in our replication set we found that only 2 of these 122 sites were differentially methylated at p<0.05, and both associations failed to pass the Bonferroni correction for testing of 122 sites. When examining years of NSAID use and pill-years of NSAID use as continuous exposures, we found 6 CpGs associated with pill-years of use which passed the FDR in the discovery set but failed to replicate. We identified 48 CpGs in the discovery set that were associated with daily use of NSAIDs in the past 12 months, but these CpGs did not reach statistical significance in the replication set with the Bonferroni correction. All CpG sites passing the FDR in the discovery set are listed in S1 Table.

**Table 2:** Differential methylation of CpG sites by NSAID use in the discovery and replication sets.

	Discovery Set		Replication Set		
	CpGs with FDR q value<0.05	N	Replicated CpGs at p<0.05	CpGs passing Bonferroi correction	N
**Breast cancer-free women only with adjustment for age**		**(N = 573)**			**(N = 181)**
Ever 'regular' NSAID use vs. Never regular NSAID use	0	(N = 355 v. 251)	-	-	
Total Years NSAID use reported	0	(N = 573)	-	-	
Total pill-years NSAID use reported	8	(N = 573)	1	0	(N = 44 v. 79)
Used an NSAID daily in the past 12 months vs. Never regular NSAID use	0	(N = 204 v. 251)	-	-	-
Used an aspirin-containing NSAID daily in the past 12 months vs. Never regular NSAID use	3	(117 v. 251)	0	-	(N = 29 v.79)
Used a non-aspirin NSAID daily in the past 12 months vs. Never regular NSAID use	0	(111 v. 251)	-	-	
**All women with adjustment for breast cancer case status and age**		**(N = 871)**			**(N = 187)**
Ever regular NSAID use vs. Never regular NSAID use	122	(N = 490 v. 381)	2	0	(N = 101 v. 86)
Total Years NSAID use reported	0	(N = 871)	-	-	
Total pill-years NSAID use reported	6	(N = 871)	0	-	
Used an NSAID daily in the past 12 months vs. non-users	48	(N = 329 v. 381)	2	0	(N = 44 v. 79)
Used an aspirin-containing NSAID daily in the past 12 months vs. non-users	0	(N = 182 v. 381)	-	-	
Used a non-aspirin NSAID daily in the past 12 months vs. non-users	0	(N = 164 v. 381)	-	-	

**Legend:** Results of robust linear regression modeling to identify differential methylation of CpG sites by NSAID use in the discovery set (N = 871). CpGs with FDR q<0.05 were considered statistically significant and were examined in a non-overlapping sample of women from the same cohort (N = 187). All models adjusted for age.

## Discussion

“Laboratory and observational studies suggest that NSAID use may influence patterns of DNA methylation in various tissues. Gastric tissue from long-term NSAID users have been shown to have differential methylation at selected genes [11], and *in vitro* NSAID treatment has been shown to alter DNA methylation in human fibroblast and lung cancer cell lines [13, 20].

Although we hypothesized that methylation differences linked to long-term NSAID use would be detectable in peripheral blood, we found no strong evidence of such an effect. No CpGs were associated with ever regularly using NSAIDs in the discovery set of women who did not subsequently develop cancer. When we expanded the discovery set to include, with adjustment, the women who subsequently developed cancer, we did find 122 CpGs that were associated with NSAIDs. Because both sets of women in the discovery set were distributed randomly to the same set of chips and batches that were adjusted for in the analysis, it seems unlikely that the change in the number of detected CpGs is due to batch, chip, or other technical effects. Nor was there evidence of insufficient adjustment or residual confounding from the case group: the coefficients of the 122 CpGs remained almost the same in the cancer-free women alone compared to cancer-free plus cases (R^2^ = 0.99). It is possible that the increased sample size resulted in increased power to detect true association, or that the CpGs detected in the expanded analysis were the result of random effects. Although we cannot formally exclude either possibility, our examination of the replication set failed to provide independent evidence for true association.

Use of a replication set provides a useful means of reducing spurious findings, but failure to replicate does not eliminate the possibility that associations with small effect size may still exist. We used Bonferroni correction for multiple testing in the replication set, and such correction may be overly conservative—yet even at a nominal threshold p value = 0.05, only 2 of the 122 sites were significant (fewer than expected by chance) and only one had the same direction of methylation effect with NSAID use. Although we have used this same replication set to confirm other small effects related to smoking [15] and participant age [21], we cannot exclude the possibility that there might be unconfirmed true positives in the discovery set. With the observed effect size of approximately 1% change in methylation seen in the discovery set, this replication sample of 187 individuals achieves between 16% and 60% power for detection using standard deviations for methylation values ranging from 0.07 to 0.03 respectively. This calculation is based on a two sample t-test with α = 0.05, and the range of standard deviations tested reflect the approximate range observed for the ten top CpGs in our comparisons. We note that a study of blood DNA from 88 men found that those taking acetylsalicylic acid had lower methylation at the ATP-binding cassette transporter A1 (*ABCA1*) gene [22] but we found no evidence for association at the 2 CpGs representing this gene on the 27K array.

It is possible that any effect of NSAID use on DNA methylation might be specific to tissues other than blood. Epigenome-wide studies conducted using DNA from tissues such as gastric epithelium, colon epithelium, and normal breast tissue may be more successful in identifying DNA methylation differences related to NSAID use. However, these tissues are difficult to obtain fresh-frozen in large numbers, which limits potential sample size. Formalin-fixed paraffin embedded (FFPE) tissue samples are more readily available than frozen tissue, and although methods are available for methylation analysis of DNA from FFPE samples, the resulting data are of lesser quality [23].

Strengths of this study include the availability of methylation array data for a large number of women with corresponding detailed NSAID exposure assessment. We were able to estimate lifetime long-term exposures to NSAID drugs including aspirin-containing and non-aspirin containing drugs. This study is substantially larger than prior studies of NSAID use and DNA methylation at candidate genes, and is the first to use epigenome-wide analysis. But we cannot exclude the possibility that small effects, or sites not covered in the 27K array, or in other tissues might still be associated with NSAID use.

## Supporting Information

S1 Table CpG sites passing FDR threshold in discovery set. CpG sites from the Illumina Infinium HumanMethylation27 beadchip array passing the FDR (q <0.05) in the discovery set of 871 women. No associations replicated with a Bonferroni correction in a second set of 187 women. CpGs marked with an asterisk(*) replicated at an unadjusted p-value of 0.05.
(DOCX)
