# Supplementary material for: Non-Steroidal Anti-Inflammatory Drug Use and Genomic DNA Methylation in Blood
Source: PLoS One. 2015 Sep 22;10(9):e0138920. doi: 10.1371/journal.pone.0138920 (PMC4578936; doi:10.1371/journal.pone.0138920)
Supplement: S1 Table — CpG sites from the Illumina Infinium HumanMethylation27 beadchip array passing the FDR (q <0.05) in the discovery set of 871 women. No associations replicated with a Bonferroni correction in a second set of 187 women. CpGs marked with an asterisk(*) replicated at an unadjusted p-value of 0.05. (DOCX) [file pone.0138920.s001.docx]

**S1 Table**. **CpG sites passing FDR threshold in discovery set.** CpG sites from the Illumina Infinium HumanMethylation27 beadchip array passing the FDR (q <0.05) in the discovery set of 871 women. No associations replicated with a Bonferroni correction in a second set of 187 women. CpGs marked with an asterisk(*) replicated at an unadjusted p-value of 0.05.

| CpG sites passing FDR threshold in discovery set | Gene | Methylation coefficient | Q value |
| --- | --- | --- | --- |
| **Breast cancer-free women only with adjustment for age** |  |  |  |
| Total pill-years NSAID use reported |  |  |  |
| cg12320676 | E2F6 | 1.10E-05 | 0.018605 |
| cg05497107 | RUFY1 | 6.59E-06 | 0.029708 |
| cg05898102 | PCDHB10 | 5.85E-05 | 0.029708 |
| cg10261589 | LOC133308 | 1.26E-05 | 0.029708 |
| cg10484485 | KIAA0922 | 1.53E-05 | 0.029708 |
| cg17804302 | NKD1 | 6.23E-06 | 0.029708 |
| cg26675382* | NUP43* | 7.75E-05* | 0.029708* |
| cg10078415 | ACSM3 | 1.88E-05 | 0.033782 |
| Used an aspirin-containing NSAID daily in the past 12 months vs. non-users |  |  |  |
| cg08375941 | GPR15 | 0.013788 | 0.04 |
| cg10891157 | CRTAP | 0.002654 | 0.04 |
| cg16957569 | INDOL1 | 0.025488 | 0.04 |
| **All women with adjustment for breast cancer case status and age** |  |  |  |
| Ever regular NSAID use vs. Never regular NSAID use |  |  |  |
| cg00234616 | TLX2 | -0.01592 | 0.015574 |
| cg03160740 | VIPR1 | -0.00871 | 0.02349 |
| cg03821311 | HIST1H1B | 0.008098 | 0.02349 |
| cg12501949 | FLJ39502 | 0.022264 | 0.02349 |
| cg15309578 | PELI1 | 0.01732 | 0.02349 |
| cg25242557 | PAX6 | -0.01019 | 0.02349 |
| cg01607495 | BAG4 | 0.011938 | 0.030107 |
| cg01796223 | CPA4 | 0.005089 | 0.030107 |
| cg11378044 | SPAG4L | 0.009099 | 0.030107 |
| cg14455991 | GPR82 | 0.024487 | 0.030107 |
| cg19740969 | CXCL9 | 0.007529 | 0.030107 |
| cg03914452 | PRR8 | 0.007753 | 0.036273 |
| cg10399228 | RAG1 | 0.016093 | 0.036273 |
| cg15984661 | CCDC8 | 0.005431 | 0.036273 |
| cg21665774 | KIAA0355 | 0.007435 | 0.036273 |
| cg01309152 | PCP4 | 0.006545 | 0.037735 |
| cg01783386 | RFT1 | 0.00241 | 0.037735 |
| cg02182354 | NEUROG2 | -0.00508 | 0.037735 |
| cg03574115 | WDR65 | 0.002572 | 0.037735 |
| cg03762535 | HAO2 | 0.010911 | 0.037735 |
| cg05538432 | C1S | 0.008666 | 0.037735 |
| cg09768051 | LRRC7 | 0.010216 | 0.037735 |
| cg10238171 | BDKRB1 | 0.015242 | 0.037735 |
| cg11154542 | CTSL | -0.009 | 0.037735 |
| cg11298412 | DKFZP564O0823 | -0.00251 | 0.037735 |
| cg13107169 | N4BP2 | 0.003175 | 0.037735 |
| cg18768283 | NUT | 0.009871 | 0.037735 |
| cg19468534 | NUSAP1 | 0.011033 | 0.037735 |
| cg21407055 | ART1 | 0.005338 | 0.037735 |
| cg23695504 | FLJ45717 | -0.01142 | 0.037735 |
| cg23751724 | C20orf133 | -0.00818 | 0.037735 |
| cg24272907 | RIMBP2 | 0.023008 | 0.037735 |
| cg24831427 | COQ3 | 0.009526 | 0.037735 |
| cg26322315 | RPA3 | 0.015703 | 0.037735 |
| cg27585441 | CCDC54 | 0.012286 | 0.037735 |
| cg05418129 | C9orf26 | 0.013199 | 0.037746 |
| cg16899442 | C16orf25 | -0.0106 | 0.037746 |
| cg17051321 | BNC1 | -0.00597 | 0.037746 |
| cg24270244 | RBM7 | -0.0006 | 0.037746 |
| cg26443244 | GDPD4 | 0.014742 | 0.037746 |
| cg27176536 | RAB26 | -0.00727 | 0.037746 |
| cg00453258 | FAM26C | 0.006497 | 0.038493 |
| cg01169726 | DLX5 | -0.00557 | 0.038493 |
| cg01637734* | CD5L* | 0.005787* | 0.038493* |
| cg02241259 | IL23R | 0.006151 | 0.038493 |
| cg02569613 | C10orf72 | -0.00956 | 0.038493 |
| cg02655623 | HSA277841 | 0.013947 | 0.038493 |
| cg03421687 | ZMYND10 | -0.00067 | 0.038493 |
| cg05965188 | KIAA0179 | 0.005455 | 0.038493 |
| cg07459489 | SLC30A8 | 0.00378 | 0.038493 |
| cg08798116 | GPC4 | 0.009593 | 0.038493 |
| cg09448875 | ABCC2 | 0.002367 | 0.038493 |
| cg09494546 | SLC16A4 | 0.004267 | 0.038493 |
| cg11152574 | ACSL5 | 0.002502 | 0.038493 |
| cg11784281 | MGC35154 | 0.004264 | 0.038493 |
| cg11808874 | FLJ14668 | 0.006486 | 0.038493 |
| cg12274479 | HSPG2 | 0.002896 | 0.038493 |
| cg13109289 | C1GALT1 | 0.004245 | 0.038493 |
| cg13328485 | ZNF650 | 0.009759 | 0.038493 |
| cg13897627 | FLJ44674 | 0.008452 | 0.038493 |
| cg15415507 | EPHA2 | -0.00832 | 0.038493 |
| cg15798530 | TRPC3 | 0.004998 | 0.038493 |
| cg16636571 | GYPB | 0.005866 | 0.038493 |
| cg16745616 | ANGPTL4 | 0.002837 | 0.038493 |
| cg16753209 | NEB | 0.009423 | 0.038493 |
| cg17653969 | SPHAR | 0.006703 | 0.038493 |
| cg19504888 | C2orf33 | 0.007854 | 0.038493 |
| cg20234959 | NID1 | 0.00677 | 0.038493 |
| cg21162961 | RRH | 0.020082 | 0.038493 |
| cg21264055 | PRR3 | -0.00658 | 0.038493 |
| cg21845297 | OTOR | 0.011025 | 0.038493 |
| cg22628694 | ZNF329 | 0.002685 | 0.038493 |
| cg23460697 | UBN1 | 0.004513 | 0.038493 |
| cg24776019 | HYPB | 0.004752 | 0.038493 |
| cg25852472 | H19 | 0.007991 | 0.038493 |
| cg11594137 | EDG3 | -0.01188 | 0.038886 |
| cg22799850 | FBXL13 | -0.01047 | 0.038886 |
| cg06501084 | KCNH5 | 0.015125 | 0.040235 |
| cg10037068 | WASPIP | 0.008447 | 0.042277 |
| cg02555579 | SLC25A18 | 0.005853 | 0.042294 |
| cg03179866 | MMP12 | 0.009181 | 0.042294 |
| cg06288351 | KCNS1 | -0.0088 | 0.042294 |
| cg06866657 | LHX6 | -0.00272 | 0.042294 |
| cg08375941 | GPR15 | 0.007364 | 0.042294 |
| cg09995854 | IL1F8 | 0.002556 | 0.042294 |
| cg10371914 | C8orf34 | 0.015407 | 0.042294 |
| cg12466095 | C14orf103 | 0.019682 | 0.042294 |
| cg15514848 | FMO1 | 0.008431 | 0.042294 |
| cg15748507 | PRLHR | -0.01458 | 0.042294 |
| cg20988616 | ENPP3 | 0.005021 | 0.042294 |
| cg23566335 | ADAM18 | 0.005622 | 0.042294 |
| cg24070292 | TPM2 | -0.00813 | 0.042294 |
| cg24921089 | AMPD3 | -0.012 | 0.042294 |
| cg16823701 | IFNA1 | 0.003819 | 0.042317 |
| cg00503840 | DLX5 | -0.00836 | 0.042363 |
| cg02602411 | PPP3R2 | 0.003962 | 0.042363 |
| cg17509872 | CNOT7 | 0.004132 | 0.042363 |
| cg05660795 | IGFBP1 | -0.00822 | 0.042486 |
| cg05898102 | PCDHB10 | 0.005639 | 0.042486 |
| cg05965402 | PI4K2B | 0.002013 | 0.042486 |
| cg09515805 | PGM2L1 | -0.00079 | 0.042486 |
| cg10748867 | C1orf33 | 0.008129 | 0.042486 |
| cg12619162 | FXYD4 | 0.005 | 0.042486 |
| cg12970008 | OSBPL5 | -0.00231 | 0.042486 |
| cg15077637 | EFTUD2 | 0.001466 | 0.042486 |
| cg19147390 | UHRF1 | 0.002421 | 0.042486 |
| cg21245652 | MAL | -0.00722 | 0.042486 |
| cg25522312 | MAD2L1 | 0.002048 | 0.042486 |
| cg24430616 | ENTPD4 | 0.004204 | 0.045036 |
| cg25763788 | HTR1B | -0.00562 | 0.045036 |
| cg15677344 | ADH1A | 0.007982 | 0.046396 |
| cg16762386 | NR0B2 | 0.004871 | 0.046396 |
| cg27550918 | SYNPO2L | 0.003666 | 0.046396 |
| cg20074593 | GPR17 | 0.004789 | 0.046496 |
| cg15013019 | LYL1 | -0.00851 | 0.047516 |
| cg01416012 | BAZ2B | 0.012794 | 0.047731 |
| cg12010995 | CYP7A1 | 0.008808 | 0.047731 |
| cg15379633 | RAB36 | -0.01861 | 0.047999 |
| cg21643191 | ABCB5 | 0.004938 | 0.048085 |
| cg09480837 | PLCE1 | 0.005726 | 0.04896 |
| cg13445249 | DSG4 | 0.011577 | 0.049638 |
| cg25662535 | ART5 | -0.0028 | 0.049638 |
| Total pill-years NSAID use reported |  |  |  |
| cg25211525 | C6orf145 | 2.21E-06 | 0.002701 |
| cg02326931 | ALG5 | 7.44E-06 | 0.032919 |
| cg05788526 | MTMR2 | 1.90E-05 | 0.032919 |
| cg14576628 | PRMT1 | 4.18E-06 | 0.032919 |
| cg15611923 | RBM8A | 6.54E-06 | 0.032919 |
| cg02121943 | TBX3 | 3.43E-05 | 0.038482 |
| Used an NSAID daily in the past 12 months vs. non-users |  |  |  |
| cg00234616 | TLX2 | -0.01834 | 0.020408 |
| cg03160740 | VIPR1 | -0.01023 | 0.025036 |
| cg03821311 | HIST1H1B | 0.009604 | 0.025036 |
| cg19468534 | NUSAP1 | 0.014037 | 0.025036 |
| cg22772878 | DIRAS1 | -0.01528 | 0.025036 |
| cg25242557 | PAX6 | -0.01147 | 0.025036 |
| cg25522312 | MAD2L1 | 0.002795 | 0.026174 |
| cg23695504 | FLJ45717 | -0.01438 | 0.026979 |
| cg24272907 | RIMBP2 | 0.027696 | 0.026979 |
| cg03716999 | FLJ46380 | 0.009504 | 0.035374 |
| cg03762535 | HAO2 | 0.012665 | 0.035374 |
| cg03914452 | PRR8 | 0.008697 | 0.035374 |
| cg05965402 | PI4K2B | 0.002703 | 0.035374 |
| cg09515805 | PGM2L1 | -0.00105 | 0.035374 |
| cg09768051 | LRRC7 | 0.012202 | 0.035374 |
| cg09972884* | RRN3* | 0.000647* | 0.035374* |
| cg12501949* | FLJ39502* | 0.023401* | 0.035374* |
| cg19740969 | CXCL9 | 0.00847 | 0.035374 |
| cg24411312 | ST14 | 0.006314 | 0.035374 |
| cg08375941 | GPR15 | 0.009305 | 0.044077 |
| cg09448875 | ABCC2 | 0.002943 | 0.044077 |
| cg00027083 | EPB41L3 | -0.02511 | 0.044347 |
| cg01637734 | CD5L | 0.006806 | 0.044347 |
| cg03251079 | HMGA2 | -0.0057 | 0.044347 |
| cg11378044 | SPAG4L | 0.009655 | 0.044347 |
| cg12179176 | SNX19 | -0.00116 | 0.044347 |
| cg15309578 | PELI1 | 0.017784 | 0.044347 |
| cg15984661 | CCDC8 | 0.005929 | 0.044347 |
| cg16745616 | ANGPTL4 | 0.003332 | 0.044347 |
| cg19837824 | NTHL1 | 0.002637 | 0.044347 |
| cg20074593 | GPR17 | 0.006046 | 0.044347 |
| cg24070292 | TPM2 | -0.01004 | 0.044347 |
| cg01607495 | BAG4 | 0.01272 | 0.045285 |
| cg11594137 | EDG3 | -0.01431 | 0.045285 |
| cg14094960 | EGFR | -0.00262 | 0.045285 |
| cg16636571 | GYPB | 0.006951 | 0.045285 |
| cg17051321 | BNC1 | -0.00675 | 0.045285 |
| cg21845297 | OTOR | 0.012595 | 0.045285 |
| cg21245652 | MAL | -0.00873 | 0.046892 |
| cg22884082 | GJB7 | 0.007111 | 0.046892 |
| cg04551925 | AQP1 | 0.005137 | 0.049399 |
| cg05840031 | PAX6 | -0.00634 | 0.049399 |
| cg06912252 | C9orf125 | -0.00073 | 0.049399 |
| cg10399228 | RAG1 | 0.016795 | 0.049399 |
| cg12025941 | RFX4 | 0.007144 | 0.049399 |
| cg19504888 | C2orf33 | 0.008983 | 0.049399 |
| cg23911465 | EIF2B2 | 0.003745 | 0.049399 |
| cg24270244 | RBM7 | -0.00067 | 0.049399 |
